# Diagnostic radiological examinations and risk of intracranial tumours in adults—findings from the Interphone Study

**DOI:** 10.1093/ije/dyab140

**Published:** 2021-10-14

**Authors:** Anssi Auvinen, Elisabeth Cardis, Maria Blettner, Monika Moissonnier, Siegal Sadetzki, Graham Giles, Christoffer Johansen, Anthony Swerdlow, Angus Cook, Sarah Fleming, Gabriele Berg-Beckhoff, Ivano Iavarone, Marie-Elise Parent, Alistair Woodward, Tore Tynes, Mary McBride, Dan Krewski, Maria Feychting, Toru Takebayashi, Bruce Armstrong, Martine Hours, Jack Siemiatycki, Susanna Lagorio, Signe Benzon Larsen, Minouk Schoemaker, Lars Klaeboe, Stefan Lönn, Joachim Schüz, and the INTERPHONE study group, Tiina Salminen, Anna Lahkola, Patricia McKinney, Naohito Yamaguchi

**Affiliations:** Faculty of Social Sciences, Unit of Health Sciences, Tampere University, Tampere, Finland; STUK—Radiation and Nuclear Safety Authority, Helsinki, Finland; Barcelona Institute of Global Health (ISGlobal), Centre for Research in Environmental Epidemiology, Universitat Pompeu Funebra, Barcelona, Spain; CIBER Epidemiologia y Salud Publica, Madrid, Spain; University of Mainz, IMBEI, Mainz, Germany; International Agency for Research on Cancer, Lyon, France; Gertner Institute, Tel Aviv, Israel; Cancer Council Victoria, Melbourne, VIC, Australia; Danish Cancer Society Research Center, Copenhagen, Denmark; Division of Genetics and Epidemiology, Institute of Cancer Research, London, UK; Division of Breast Cancer Research, Institute of Cancer Research, London, UK; School of Population and Global Health, University of Western Australia, Crawley, WA, Australia; University of Leeds, Leeds, UK; Faculty of Public Health, University of Bielefeld, Germany; Istituto Superiore di Sanità, Rome, Italy; INRS Centre Armand-Frappier Santé Biotechnologie, Institut National de la Recherche Scientifique, Université du Québec, Laval, QC, Canada; School of Population Health, University of Auckland, Auckland, New Zealand; National Institute of Occupational Health, Oslo, Norway; British Columbia Cancer Agency, Vancouver, BC, Canada; McLaughlin Centre for Population Health Risk Assessment, University of Ottawa, ON, Canada; Karolinska Institutet, Stockholm, Sweden; Keio University School of Medicine, Tokyo, Japan; School of Public Health, University of Sydney, Sydney, NSW, Australia; Université Lyon 1, IFSTTAR, UMRESTTE, Bron, France; University of Montreal, Montreal, QC, Canada; Istituto Superiore di Sanità, Rome, Italy; Danish Cancer Society Research Center, Copenhagen, Denmark; Division of Genetics and Epidemiology, Institute of Cancer Research, London, UK; Norwegian Radiation Protection Authority, Østerås, Norway; Karolinska Institutet, Stockholm, Sweden; Region Halland, Research and Development, Sweden; International Agency for Research on Cancer, Lyon, France

**Keywords:** Radiation, ionizing, glioma, meningioma, neuroma, acoustic, case-control studies

## Abstract

**Background:**

Exposure to high doses of ionizing radiation is among the few well-established brain tumour risk factors. We used data from the Interphone study to evaluate the effects of exposure to low-dose radiation from diagnostic radiological examinations on glioma, meningioma and acoustic neuroma risk.

**Methods:**

Brain tumour cases (2644 gliomas, 2236 meningiomas, 1083 neuromas) diagnosed in 2000–02 were identified through hospitals in 13 countries, and 6068 controls (population-based controls in most centres) were included in the analysis. Participation across all centres was 64% for glioma cases, 78% for meningioma cases, 82% for acoustic neuroma cases and 53% for controls. Information on previous diagnostic radiological examinations was obtained by interviews, including the frequency, timing and indication for the examinations. Typical brain doses per type of examination were estimated based on the literature. Examinations within the 5 years before the index date were excluded from the dose estimation. Adjusted odds ratios were estimated using conditional logistic regression.

**Results:**

No materially or consistently increased odds ratios for glioma, meningioma or acoustic neuroma were found for any specific type of examination, including computed tomography of the head and cerebral angiography. The only indication of an elevated risk was an increasing trend in risk of meningioma with the number of isotope scans, but no such trends for other examinations were observed. No gradient was found in risk with estimated brain dose. Age at exposure did not substantially modify the findings. Sensitivity analyses gave results consistent with the main analysis.

**Conclusions:**

There was no consistent evidence for increased risks of brain tumours with X-ray examinations, although error from selection and recall bias cannot be completely excluded. A cautious interpretation is warranted for the observed association between isotope scans and meningioma.

Key Messages

- Medical diagnostic radiation is a major source of ionizing radiation exposure.
- Various brain tumours can be induced by exposure to ionizing radiation, and several studies have suggested increased risks from low doses of medical diagnostic radiation.
- We evaluated the risk of glioma, meningioma and acoustic neuroma in relation to self-reported diagnostic radiation exposure in a large international collaborative case-control study.
- We found no clear or systematic increased risks of glioma, meningioma or acoustic neuroma related to medical imaging, though patients with glioma may underestimate their exposure, thereby biasing the results

## Introduction

The average age-standardized incidence rates (world standard population) of brain and nervous system cancer are approximately five per 100 000 for men and four for women in countries with population-based cancer registries (countries with high developmental index).^1^ Most cancer registries do not, however, record benign brain tumours, including meningiomas and acoustic neuromas. The reported incidence of meningiomas has been two to four per 100 000 for women and less than two per 100 000 for men in the Nordic countries.^2^

The aetiology of brain tumours is largely unknown. Some rare hereditary syndromes, including tuberous sclerosis for glioma and von Hippel-Lindau syndrome for acoustic neuroma, carry a very high risk, but they account only for a tiny fraction of all cases. High doses of ionizing radiation (several Gray) increase the risk of glioma and meningioma in radiotherapy patients.^3^ In addition, dose-response has been shown for all main brain tumour types among atomic bomb survivors.^4^ In industrialized countries, medical uses of radiation comprise most of the population’s whole-body radiation dose, including radiation dose to the brain.^5^ Several studies have reported an association between dental X-rays and meningioma risk,^6–15^ with some suggestions also for glioma^8–9^ and acoustic neuroma,^16^ but few have been adequately powered, evaluated medical diagnostic radiography comprehensively, or estimated the radiation dose.

We analysed the risk of glioma, meningioma and acoustic neuroma related to previous diagnostic X-ray examinations using the Interphone study, a large international case-control study of brain tumours.^17^

## Methods

Ethical committee review was conducted according to local regulation and all participants signed a written informed consent before participation. The study procedures followed the principles of the Declaration of Helsinki.

The Interphone study is an international collaborative case-control study of brain tumours, which has collected data from 16 centres in 13 countries. The primary goal was to evaluate the possible risk related to the use of mobile phones, but information on several other potential risk factors was also collected.^17–19^

The eligibility criteria for cases included age 30–59 years at diagnosis and diagnosis of a glioma, meningioma or acoustic neuroma with histological or unequivocal radiological confirmation. The study period for eligible diagnoses ranged 2–4 years during 2000–04, slightly varying by country.^17^ The cases were ascertained from neurosurgery or neurology departments to ensure rapid enrolment after diagnosis, and at some centres acoustic neuromas also from cancer registries or ear, nose and throat clinics.

Controls were identified from local population rosters (such as electoral rolls), with frequency or individual matching by age (within 5-year age groups), sex and area of residence. For glioma and meningioma, a 1:1 case-control matching ratio was used, except for 1:2 in Germany. For acoustic neuroma, the matching ratio was generally 1:2. *Post* *hoc* matching was used to construct case-control pairs for centres with frequency matching, with one control selected for each case so that an index case could be assigned for the control based on the case’s date of diagnosis (except that two controls were assigned to acoustic neuroma cases, and all cases in Germany).

Whenever possible, consenting subjects were interviewed face-to-face by trained interviewers using computer-assisted personal interview (CAPI) software for collecting exposure information. If the subject had died or was too ill to participate, a proxy respondent was interviewed where this was possible and permitted by the ethical committee.^17^ The median time from diagnosis to interview was 3 months for glioma and meningioma and 6 months for acoustic neuroma.^17^ The interviews covered, among many other aspects, lifetime history of exposure to medical uses of radiation, for both diagnostic and therapeutic purposes, as well as occupational exposure to ionizing radiation. Details of radiological examinations of the head and neck region were obtained. The types of diagnostic X-ray examination included those of the skull, nose, jaw, facial bones, sinuses and neck, as well as dental X-rays as separate entries. Computed tomograms (CT scans), angiograms, sialograms and isotope scans (nuclear medicine) were covered in similar detail. Head CT covered scans of the brain, skull and facial bones, orbits and paranasal sinuses. For each examination type (except dental X-rays other than full mouth), lifetime number of examinations was recorded and for each examination, information was sought on age at first exposure and reason (clinical indication) for the procedure. For dental radiography other than full mouth X-ray, participants were asked about frequency of examinations (annually, every 2–3 years, at least every 5 years, less frequently, irregularly or none) rather than numbers. Diagnostic examinations related to the detection of the brain tumour were excluded from the analysis, as well as other examinations during the 5 years preceding the index date (date of diagnosis for cases and that of the case for the matched controls). Patients with a history of radiotherapy preceding the index date were excluded from the analyses of diagnostic radiography (28 from glioma, 140 from meningioma and 18 from neuroma analysis). In addition, information on mobile phone use, sociodemographic factors, lifestyle, occupational exposures, medical history and family history of cancer was collected.

Brain dose was estimated for each examination type from the literature (mainly the International Commission on Radiological Protection, United Nations Scientific Committee on the Effects of Ionizing Radiations, UNSCEAR and National Radiological Protection Board reviews).^20–24^ Confirmatory dose calculations for the most common X-rays were performed using PCXMC software^25^ and based on settings (voltage and tube current time) provided in the *WHO Manual for Diagnostic Imaging*.^26^ The brain doses from the most common examinations are relatively low, 0.1–1.4 mGy, and they were regarded as low-dose examinations (Table 1). As doses from CT and fluoroscopic procedures range 2–40 mGy, they were classified as high-dose examinations and analysed separately. In addition, analyses were performed in relation to indicative cumulative dose estimates (dose index), based on the typical doses to the brain by examination type and period. For isotope scans (nuclear medicine), we covered all examination types, not only brain scans. The interviews did not specify isotopes or amount of activity used, as patients are unlikely to be aware of such details. Instead, standard procedures described in protocols and guidelines were assumed to have been used. Changes over time were incorporated in dose calculations, with dose estimates for 1990 onward shown in Table 1 and correction factors applied for earlier exposures (for radiography in the 1980s a factor of 1.5, in the 1970s 2 and in the 1960s 2.5, based on Linet *et al.* 2009,^27^ UNSCEAR 2008^21^ and Melo *et al*. 2016^23^; for CT, a correction factor of 0.2 before 1980 and 0.5 for 1980–89, based on UNSCEAR^21^; and for nuclear medicine scans, a factor of 2 before 1990, based on Linet *et al.* 2009^27^). A summary of the dose estimates employed and frequencies of diagnostic examinations (together with indication where available) by case/control status and tumour type is given in Supplementary Table S1, available as Supplementary data at *IJE* online.

**Table 1:** Estimated typical brain dose from head and neck X-rays in the 1990s. For earlier exposures, correction factors were applied to account for changes in patient dose over time: for radiography in the 1980s, a correction factor of 1.5, in the 1970s a factor of 2 and in the 1960s of a factor of 2.5; for CT, a factor of 0.2 before 1980 and of 0.5 for 1980–89; for nuclear medicine scans, a factor of 2 before 1990

X-ray examination type	Estimated brain dose (mGy)^a^
Skull	0.9^a^
Neck/cervical spine	0.1
Full mouth	0.01
Other dental	0.002
Cerebral angiography	5
Sialography	2
Computed tomography of the head	20
Computed tomography of the neck	1
Thyroid isotope scan	1^b^
Other isotope scan	2^c^

a Includes X-rays of the skull, sinuses, facial bones, jaw, nasal bones. Dose range depending on indication from 0.01 mGy for orthodontic treatment to 1.0 mGy for trauma/road accident.

b Calculated for I-131 and Tc-99m.

c Calculated for Tc-99m for lung and bone scans, I-131 for kidney, Tl-201 for heart. Dose range from 0.2 mGy for a lung scan to 3 mGy for a heart scan.

The data were analysed using conditional logistic regression. In analyses of glioma and meningioma, we adjusted for education and self-reported history of allergies; for acoustic neuroma, additional adjustments for smoking and for occupational and leisure time exposure to loud noise were made. Exposure indicators used in the analyses included specific type of X-ray examination, low-dose examinations combined (typical brain dose <1 mGy), high-dose examinations (at least 1 mGy) and cumulative dose index (0–1, 1–10, 10–30 and 30+ mGy). There were too few exposed cases for separate analyses of frequencies of cerebral angiography or sialography. Subjects who had undergone high-dose examinations were excluded from analyses of low-dose examinations, and dose from low-dose examinations was ignored (but the subjects included) in the combined analysis of all high-dose examinations. Analyses were also conducted by age at exposure. An alternative analysis of the number of low-dose examinations was carried out excluding all dental X-rays, with the rationale that they had the lowest doses and were likely to have had the most uncertainty in reporting due to their high frequency.

Sensitivity analyses were carried out excluding: subjects reporting occupational exposure to ionizing radiation (150 glioma cases and 194 controls, 110 meningioma cases and 130 controls, 35 neuroma cases and 126 controls); subjects with poor quality of information as assessed by interviewers (153 glioma cases and 172 controls, 87 meningioma cases and 102 controls); those with proxy interviews (335 glioma cases and 362 controls, 47 meningioma cases and 51 controls); and those reporting a diagnosis of tuberous sclerosis or neurofibromatosis (12 glioma cases and 13 glioma controls, 11 meningioma cases and 11 meningioma controls). Another alternative analysis of the cumulative dose was carried out with a higher dose assigned to head CT (40 mGy instead of 20).

## Results

The Interphone study recruited 6311 cases with intracerebral tumours and 7658 controls from 13 countries (Denmark, Finland, Norway, Sweden, Germany, United Kingdom, France, Italy, Israel, Japan, Canada, Australia and New Zealand). Participation was 64% for glioma cases, 78% for meningioma cases, 82% for acoustic neuroma cases and 53% for controls. A total of 14 095 participants provided information about diagnostic medical examinations. Of them, 12 021 subjects were included in analysis of the ionizing radiation dose from radiological examinations and risk of brain tumours: 2644 gliomas, 2236 meningiomas and 1083 acoustic neuromas, with 6068 controls.

Low-dose examinations were frequently reported: 60% or more for each group for dental radiography, followed by skull X-ray ranging 16–20% (Table 2). High-dose examinations were considerably less common: head CT 5–9% and isotope scans 3–8%. The frequencies of various examinations were largely comparable for glioma and meningioma cases, whereas low-dose examinations tended to be more common for acoustic neuroma cases.

**Table 2:** Exposure to radiological diagnostic examinations among cases and controls, by tumour type (excluding examinations within 5 years before the diagnosis date in cases and reference date in controls)

	Glioma, *n* (%)		Meningioma, *n* (%)		Acoustic neuroma, *n* (%)	
X-ray examination	Cases	Controls	Cases	Controls	Cases	Controls
Skull	425 (16)	579 (20)	364 (16)	411 (17)	201 (19)	368 (18)
Neck	217 (8)	318 (11)	258 (12)	269 (11)	133 (12)	222 (11)
Full mouth	351 (13)	448 (16)	378 (17)	411 (17)	191 (18)	334 (16)
Other dental	1663 (63)	1833 (63)	1334 (60)	1444 (59)	776 (72)	1336 (64)
Any low-dose examination^a^	1791 (68)	1990 (69)	1466 (66)	1555 (63)	841 (78)	1455 (70)
Computed tomography of the head	145 (5)	193 (7)	192 (9)	212 (9)	79 (7)	157 (8)
Computed tomography of the neck	25 (1)	31 (1)	8 (<1)	11 (<1)	2 (<1)	10 (<1)
Cerebral angiography	19 (1)	24 (1)	19 (1)	31 (1)	9 (1)	18 (1)
Sialography	7 (<1)	8 (<1)	12 (1)	11 (<1)	4 (<1)	10 (<1)
Isotope scan	92 (3)	135 (5)	159 (7)	186 (8)	38 (4)	70 (3)
Any high-dose examination^b^	255 (10)	342 (12)	339 (15)	404 (16)	120 (11)	240 (12)
Total	2644 (100)	2888 (100)	2236 (100)	2460 (100)	1083 (100)	2086 (100)

a Radiographic examinations listed above.

b CT, fluoroscopy and isotope examinations.

In analyses of low-dose and high-dose examinations in relation to risk of glioma, the odds ratio (OR) estimates were mostly below unity, often substantially so (Table 3). No trends of increasing OR estimates were observed in relation to increasing frequency of examinations.

**Table 3:** Odds ratios (ORs with 95% confidence intervals, CIs) of brain tumours in relation to diagnostic X-ray examinations, with numbers of exposed cases and controls

Examination type	Glioma			Meningioma		Acoustic neurinoma		
	Number of exposed cases/controls		OR (95% CI)	Number of exposed cases/controls	OR (95% CI)	Number of exposed cases/controls	OR (95% CI)	
**Skull X-ray**								
Any vs none	425/579		0.72 (0.62-0.83)	364/411	0.89 (0.75-1.06)	201/368	0.97 (0.78-1.20)	
No. of examinations (reference none)								
1-2	360/489		0.72 (0.62-0.84)	282/339	0.85 (0.71-1.02)	179/304	1.03 (0.83-1.29)	
3-4	42/61		0.68 (0.45-1.02)	47/52	0.91 (0.60-1.38)	16/44	0.67 (0.37-1.22)	
5 or more	23/29		0.76 (0.44-1.32)	35/20	1.81 (1.01-3.26)	6/20	0.60 (0.24-1.55)	
**Neck X-ray**								
Any vs none	217/318		0.70 (0.58-0.84)	258/269	1.03 (0.85-1.25)	133/222	1.05 (0.83-1.34)	
No. of examinations (reference none)								
1-2	192/273		0.71 (0.58-0.87)	223/236	1.01 (0.83-1.24)	115/196	1.02 (0.79-1.31)	
3-4	15/34		0.48 (0.26-0.89)	28/24	1.26 (0.72-2.19)	14/16	1.62 (0.77-3.37)	
5 or more	10/11		1.05 (0.44-2.51)	7/9	0.84 (0.31-2.27)	4/10	0.80 (0.24-2.66)	
**Full mouth X-ray**								
Any vs none	351/448		0.85 (0.72-1.00)	378/411	0.97 (0.82-1.16)	191/334	1.00 (0.80-1.23)	
No. of examinations (reference none)								
1-2	303/393		0.84 (0.71-1.00)	324/358	0.96 (0.80-1.14)	164/287	1.00 (0.80-1.26)	
3-5	42/38		1.17 (0.75-1.84)	39/36	1.15 (0.73-1.83)	16/35	0.75 (0.40-1.41)	
5 or more	6/17		0.39 (0.15-0.99)	15/17	1.00 (0.49-2.04)	11/12	1.52 (0.62-3.73)	
**All low-dose examinations** (participants with any high-dose examinations excluded)								
Any vs none	1791/1990		0.69 (0.58-0.83)	1466/1555	0.84 (0.67-1.05)	841/1455	1.21 (0.92-1.61)	
No. of examinations (reference none)								
1-2	1449/1488	0.74 (0.62-0.89)		1101/1143	0.85 (0.68-1.07)	650/1135	1.20 (0.90-1.59)	
3-4	235/360		0.51 (0.40-0.65)	241/290	0.77 (0.59-1.01)	139/227	1.33 (0.94-1.88)	
5 or more	107/142		0.59 (0.43-0.81)	124/122	0.94 (0.67-1.31)	52/97	1.15 (0.74-1.78)	
**Computed tomography of the head**								
Any vs none	145/193		0.83 (0.66-1.04)	192/212	1.06 (0.86-1.31)	79/157	0.97 (0.72-1.31)	
No. of examinations (reference none)								
1	109/155		0.77 (0.60-1.00)	155/163	1.10 (0.87-1.38)	65/129	0.98 (0.71-1.35)	
2	21/28		0.83 (0.47-1.48)	20/39	0.59 (0.34-1.03)	10/20	1.04 (0.47-2.27)	
3 or more	15/10		1.67 (0.74-3.77)	17/10	2.35 (1.06-5.21)	4/8	0.72 (0.20-2.58)	
**Computed tomography of the neck**								
Any vs none	25/31		1.02 (0.60-1.75)	8/11	0.84 (0.33-2.12)	2/10	0.32 (0.07-1.49)	
No. of examinations (reference none)								
1	19/19		1.29 (0.68-2.47)	4/10	0.49 (0.15-1.57)	2/7	0.45 (0.09-2.26)	
2	4/9		0.52 (0.16-1.72)	4/1	4.12 (0.44-38.5)^a^	−/3	NE	
3 or more	2/3		0.88 (0.14-5.44)	−/-	NE	−/-	NE	
**Cerebral angiography**								
Any vs. none	19/24		0.99 (0.53-1.83)	19/31	0.79 (0.44-1.42)	9/18	0.87 (0.39-1.97)	
**Sialography**								
Any vs none	7/8		0.99 (0.36-2.76)	12/11	1.33 (0.58-3.06)	4/10		1.06 (0.33-3.46)
**Isotope scans**								
Any vs none	92/135		0.98 (0.73-1.31)	159/186	1.32 (1.03-1.70)	38/70		0.99 (0.65-1.52)
No. of examinations (reference none)								
1	65/99		0.94 (0.67-1.31)	96/127	1.13 (0.84-1.52)	29/54		0.97 (0.60-1.58)
2	17/24		1.01 (0.52-1.96)	38/39	1.62 (1.01-2.59)	5/12		0.80 (0.27-2.42)
3 or more	10/12		1.28 (0.55-3.03)	25/20	2.17 (1.18-4.02)	4/4		1.93 (0.47-7.93)
**All high-dose examinations** (low-dose examinations excluded)								
Any vs none	255/342		0.90 (0.75-1.07)	339/404	1.08 (0.91-1.28)	120/240		0.94 (0.74-1.21)
No. of examinations (reference none)								
1	165/232		0.84 (0.68-1.04)	217/271	0.98 (0.81-1.19)	90/179		0.95 (0.72-1.25)
2	53/74		0.90 (0.62-1.31)	66/85	1.10 (0.78-1.55)	19/40		0.91 (0.52-1.60)
3 or more	37/36		1.32 (0.82-2.12)	56/48	1.72 (1.15-2.58)	11/21		0.95 (0.43-2.08)

NE, not estimable.

a Estimate given for 2 or more examinations, categories collapsed due to low frequencies for 3+ examinations.

For meningioma, the ORs were generally close to unity (Table 3). An increased OR was related to five or more skull X-rays [1.81, 95% confidence interval (CI) 1.01-3.26], though without a trend across the categories. ORs below unity were found for any past dental X-ray, as well as for regular dental X-rays (not including full mouth X-rays). Three or more head CTs were also associated with an elevated meningioma risk (OR 2.35, 95% CI 1.06-5.21). An increased OR was also observed in relation to any isotope scan vs none (1.32, 95% CI 1.03-1.70). In high-dose examinations, an increased OR was observed for two isotope scans (1.62, 95% CI 1.01-2.59) and three or more isotope scans (2.17, 95% CI 1.18-4.02), with a trend of increasing OR with increasing number of examinations. Moreover, among all high-dose examinations an increased OR was related to three or more such examinations (1.72, 95% CI 1.15-2.58). No increased ORs for any X-ray examination type were found for acoustic neuroma (Table 3).

Analysis by typical dose level, combining the radiation exposure from different types of X-ray examinations, showed no monotonic increase in risk of any brain tumour type (Table 4). We found no differences by age at first exposure for any of the tumour types, when comparing exposures from examinations conducted before versus after age 20 years (results not shown). However, numbers of cases exposed at young age were small (20 gliomas, 19 meningiomas, nine neuromas).

**Table 4:** Odds ratio, OR (with 95% confidence interval, CI) of brain tumour by cumulative dose index for diagnostic radiography, by tumour type and age at exposure, with numbers of exposed cases and controls

Dose index^a^	Glioma		Meningioma		Acoustic neuroma	
	Cases/controls	OR (95% CI)	Cases/controls	OR (95% CI)	Cases/controls	OR (95% CI)
0	424/319	1 (reference)	239/212	1 (reference)	99/214	1 (reference)
<1 (0.1)	1578/1708	0.69 (0.58-0.82)	1319/1446	0.85 (0.69-1.05)	696/1273	1.24 (0.94-1.63)
1-9 (3)	487/653	0.59 (0.48-0.72)	473/571	0.83 (0.66-1.05)	208/442	1.06 (0.78-1.44)
10-29 (18)	123/178	0.54 (0.41-0.72)	168/194	0.88 (0.66-1.17)	67/131	1.16 (0.78-1.73)
30 or more (55)	32/30	0.86 (0.51-1.45)	37/37	1.07 (0.64-1.77)	13/27	1.01 (0.47-2.16)
Medical exposure at ages 0-19 years only^b^						
0	1443/1561	1 (reference)	1340/1393	1 (reference)	572/1093	1 (reference)
<1 (<0.1)	996/1024	1.02 (0.90-1.17)	750/873	0.82 (0.71-0.94)	430/802	1.03 (0.86-1.23)
1-9 (3)	192/285	0.75 (0.61-0.92)	132/184	0.74 (0.58-0.95)	72/179	0.74 (0.54-1.01)
10 or more (17)	13/18	0.76 (0.37-1.57)	14/10	1.39 (0.59-3.30)	9/12	1.32 (0.53-3.26)

a Estimate of typical brain dose calculated from all reported medical diagnostic procedures as an indicator of cumulative organ dose (mGy), in brackets category-specific mean dose index for glioma [similar also for meningioma and neurinoma with some exceptions: for all ages, the mean 51 mGy for meningioma and 46 mGy for acoustic neuroma in the highest (30+) category, and for ages <20 years, the mean 15 mGy for neuroma].

b Not enough exposed subjects to analyse higher exposures (one glioma, two meningiomas, no neuromas in 30+ group).

Adjustment for potential confounders (education and allergies for all tumour types, also smoking and loud noise for neuromas) had only a marginal effect on the results, although it tended generally to increase slightly the risk estimates for glioma and meningioma, with no consistent direction for acoustic neuroma.

In sensitivity analyses, excluding cases and controls with occupational exposure, those with proxy interviews or with poor compliance at interview had little or no impact on the risk estimates for number of high-dose examinations in glioma and meningioma. Restricting the analysis to case-control pairs with similar level of education did not reveal a clear gradient by dose index or number of examinations (whether continuous or categorical) for any tumour type. Likewise, the results were not materially affected when subjects with fluoroscopy (with highly uncertain dose estimates), occupational exposure to ionizing radiation or with proxy interviews were excluded, or a higher dose index was used for head CT, or lower correction factors were used for radiographic examinations before 1990. When dental X-rays were excluded from the analysis of low-dose examinations, the risk estimate for subjects with five or more examinations increased (from 0.94 to 1.48), but was still imprecise for meningioma, and the point estimate remained below unity for glioma (0.59 to 0.74).

## Discussion

Our results do not indicate increased risks of glioma, meningioma or acoustic neuroma associated with common diagnostic radiological examinations. Some increased ORs were observed only for meningioma, and the only clearly increasing gradient with number of specific examinations (out of three specific low-dose and three high-dose examinations evaluated) was observed between number of isotope scans and meningioma, with no consistent finding for an analysis pooling all high-dose examinations. This finding requires confirmation by other studies, given that it may be a chance finding owing to multiple comparisons arising from evaluation of risks of three tumour types for nine types of radiological examination. The plausibility of the finding would be improved if isotope scans delivered high doses compared with other examinations, but the doses are likely to be lower than in head CT (which had comparable frequency in our sample). No similar association with isotope scans was observed for glioma or acoustic neuroma.

We found no increased risks of meningioma from dental radiography. This contrasts with a number of earlier studies suggesting such an association.^7–10^^,^^12–14^ For glioma, most studies have shown no association with dental^10^^,^^14^^,^^28–30^ or other head X-rays,^8^^,^^31^ with some exceptions.^32^ However, the dose to the brain in dental radiography is very small (<0.01 mGy). Applying the quantitative risk estimates from atomic bomb survivor studies (excess rate ratio 1.5–1.8 per Gy for schwannoma, glioma and meningioma) to this dose level suggests an expected magnitude of risk smaller than would be detectable in any epidemiological study (rate ratio < 1.0001).^4^

Far fewer studies have evaluated risks related to other head X-rays, despite higher doses. They have not shown elevated risk for gliomas,^31^ but some indications of risk increases for meningioma and schwannoma.^11^^,^^33^^,^^34^

For glioma, frequencies of reported radiological examinations were lower for cases than controls and consequently most risk estimates were below unity. Compelling evidence from previous studies indicates increased risks from ionizing radiation,^3^^,^^4^^,^^35^^,^^36^ and therefore this very likely represents bias and not a true biological effect, i.e. reduction in risk. The dose levels are at least three orders of magnitude smaller than those, for which any therapeutic effect through manifest cell killing could be achieved.^37–38^

Several sources of error need to be considered in the interpretation of our findings, including the ORs consistently below unity for gliomas. Information bias in terms of differential completeness of recall by cases and controls is of concern. Classically, cases tend to report their exposures more completely than controls and this may be applicable particularly to meningioma and acoustic neuroma. Recall bias may apply even more to examinations in distant past, such as during childhood. These are of particular importance, as radiation exposure in childhood bears higher risk than in adulthood. In previous studies, incomplete reporting of diagnostic radiological examination has been shown, with lower completeness for controls than thyroid cancer cases, though without material bias in the risk estimates.^39–40^ No major difference in completeness of reporting past diagnostic X-rays was found between patients with parotid tumours and controls.^41^ Symptoms of glioma, on the other hand, may affect memory and cognitive function both physiologically and psychologically, i.e. the tumour can impair brain function, possibly affecting the ability to concentrate at interview.^42–43^ Reduced ORs were mainly found for glioma and not meningioma or acoustic neuroma, which is consistent with this possibility.

Selection bias can also distort results of any case-control study. In our study, participation by controls was 53%. In this study, as well as more generally, higher participation has been reported by people with higher socioeconomic status, higher level of education and/or higher income levels.^44^^,^^45^ Given that the use of health care services or even specific examinations may be more common in these groups (who may, for instance, be likely to hold private health insurance), frequency of X-rays among the enrolled controls may be higher than in the population in general, which may overestimate the exposure levels in the base population and thereby underestimate true odds ratios. This could at least partly account for the frequent odds ratios below unity in our results, as well as the U-shaped exposure-outcome gradient in the analysis by typical brain dose by examination type. However, adjustment for education as an indicator of socioeconomic status (SES) increased the risk estimates only marginally in glioma and meningioma, without a systematic effect in neurinoma. Restricting the analysis to case-control pairs with similar education did not appreciably affect the results.

As mentioned above, chance and statistical power also need to be considered. Despite an exceptionally large study population overall, we had only small numbers of several specific examinations, particularly those involving high doses or examinations before adulthood. Whereas this reduces the statistical power to detect an association, if any, it also means that if there was a risk, the fraction of brain tumours attributable to diagnostic examinations would most likely be small due to the low exposure prevalence.

We collected information on established risk factors for brain tumours, including hereditary syndromes, allergies and high-dose radiation exposures. We were able to control for their effect by adjustment or exclusion, which likely minimized any confounding due to them. Additionally, sensitivity analyses excluding those with occupational radiation exposure or those with the most uncertainty in exposure assessment (poor compliance at interview, or proxy interview) yielded results that were consistent with the main analysis. This suggests that these issues did not have a major influence on our results.

We were unable to collect detailed information about individual X-ray examinations, to identify the precise examination type or obtain information on the radiography settings used. Instead, crude brain dose estimates were constructed to represent typical exposures. These should, therefore, not be regarded as accurate numerical values, but semi-quantitative estimates with substantial uncertainty. They allow combining radiation exposures from several examination types into an index of overall exposure, which we regard as an improvement compared with earlier studies. Nevertheless, such estimates cannot be treated as accurate doses, which require physical measures of the amount of radiation exposure. Therefore, we chose not to present any numerical risk estimates per dose unit.

For several X-ray examinations, the radiation dose is not homogeneous across the entire brain, but the beam is limited to a part of it (e.g. primarily the lower part of the brain is affected in neck examinations). This is likely to cause non-differential misclassification, as the dose varies by tumour location and type (intracerebral glioma, meningioma adjacent to the skull and acoustic neuroma at the base of the skull). Such dose heterogeneity is likely to induce exposure misclassification, which could dilute any true effects if non-differential.

In conclusion, we found no increased risks of brain tumours associated with diagnostic radiography, except for previous isotope scans showing an association with risk of meningioma. This association should be evaluated in future studies to assess whether the result represents a true effect or if it is a chance finding. Overall, interview-based information on diagnostic radiography has limitations, and ideally other exposure assessment methods, such as records of examinations from radiology departments, are preferable.

## Supplementary data

Supplementary data are available at *IJE* online.

## Funding

This work was financially supported in European countries and Israel by the European Union Fifth Framework Program, ’‘Quality of Life and Management of Living Resources’’ [contract QLK4-CT-1999901563] and the International Union against Cancer (UICC). The UICC received funds it disbursed for this purpose from the Mobile Manufacturers’ Forum and the GSM Association. Provision of funds to the INTERPHONE study investigators via the UICC was governed by agreements that guaranteed INTERPHONE’s complete scientific independence. The terms of these agreements are publicly available at [http://www.iarc.fr/ENG/Units/RCAd.html/]. The study in Australia was funded by the National Health and Medical Research Council of Australia and supported by in-kind provision of infrastructure necessary to the study by Cancer Council NSW and Cancer Council Victoria. The Canadian centres in Ottawa/Vancouver were supported by a university–industry partnership grant from the Canadian Institutes of Health Research (CIHR), the latter including partial support from the Canadian Wireless Telecommunications Association. The CIHR university–industry partnerships programme also includes provisions that ensure complete scientific independence of the investigators. D.K. is the Natural Sciences and Engineering Research Council of Canada Chair in Risk Science at the University of Ottawa. The Canada-Montreal study was primarily funded by a grant from the Canadian Institutes of Health Research [project 15 MOP-42525]. Additionally, J.S.’s research team was partly funded by the Canada Research Chair programme and by the Guzzo-CRS Chair in Environment and Cancer. M-E.P. had a salary award from the Fonds de la recherche en santé du Québec. The Japanese Interphone study was funded by the Ministry of Internal Affairs and Communications of Japan. The conduct of the study in New Zealand was supported by the Health Research Council of New Zealand; the Cancer Society of New Zealand; the Wellington Medical Research Foundation; the Hawke’s Bay Medical Research Foundation; and the Waikato Medical Research Foundation. The Finnish Interphone study was supported by Academy of Finland [Grant No 80921 and 48757] and Emil Aaltonen Foundation.

## Data availability

The data underlying this article cannot be shared publicly due to privacy regulation with different requirements in different countries. The data will be shared to the extent possible upon reasonable request to the study coordinator at the International Agency for Research on Cancer (contact: Joachim Schüz).

## Author contributions

Study design E.C., J.S., A.A. M.F., S.S., G.G., C.J., A.S., A.C., S.F., I.I., M-E.P., A.C., S.F., A.W., T.T., M.Mc., D.K., M.F., T.T., B.A., M.H., J.S. Data collection: all authors; Project planning and management: E.C., S.S., M.B., J.S., A.A. Data analysis: A.A., M.Mo. Preparing the first manuscript version, editing, revision and responses to comments: A.A. Manuscript editing, revision and approval of the final version: all authors. The INTERPHONE study group also includes: Tiina Salminen (Finland), Anna Lahkola (Finland), Patricia McKinney (UK North), Prof. Naohito Yamaguchi (Japan). Where authors are identified as personnel of the International Agency for Research on Cancer/World Health Organization, the authors alone are responsible for the views expressed in this article and they do not necessarily represent the decisions, policy or views of the International Agency for Research on Cancer/World Health Organization.

## Conflict of interest

None declared.

## Supplementary Material

dyab140_Supplementary_Data
