# Corrigendum to: Diagnostic radiological examinations and risk of intracranial tumours in adults—findings from the interphone study

**DOI:** 10.1093/ije/dyab240

**Published:** 2021-11-25

**Authors:** Anssi Auvinen, Elisabeth Cardis, Maria Blettner, Monika Moissonnier, Siegal Sadetzki, Graham Giles, Christoffer Johansen, Anthony Swerdlow, Angus Cook, Sarah Fleming, Gabriele Berg-Beckhoff, Ivano Iavarone, Marie-Elise Parent, Alistair Woodward, Tore Tynes, Mary McBride, Dan Krewski, Maria Feychting, Toru Takebayashi, Bruce Armstrong, Martine Hours, Jack Siemiatycki, Susanna Lagorio, Signe Benzon Larsen, Minouk Schoemaker, Lars Klaeboe, Stefan Lönn, Joachim Schüz, and the INTERPHONE study group

First published online: 14 October 2021, *Int J Epidemiol* 2022;**51**:537–46, https://doi.org/10.1093/ije/dyab140

In the originally published version of this manuscript, an author’s name was incorrect.

The author’s name should read “Maria Feychting” instead of “Maria Feyching”.

This error has been corrected.

