# Supplementary material for: Diagnostic radiological examinations and risk of intracranial tumours in adults—findings from the Interphone Study
Source: Int J Epidemiol. 2021 Oct 14;51(2):537–46. doi: 10.1093/ije/dyab140 (PMC9082802; doi:10.1093/ije/dyab140)
Supplement: dyab140_Supplementary_Data [file dyab140_supplementary_data.docx]

SUPPLEMENTARY TABLE S1: Indicative brain dose assigned by type of x-ray examination using 5-year lag and excluding participants with prior radiotherapy with numbers of exposed cases and controls

| **X-ray examination** | **Type/Indication** | **Brain dose (mGy)** | **Cases, N (%)** | **Controls, N (%)** |
| --- | --- | --- | --- | --- |
| Head^1^ | Headache | 0.9 | 190 (1.5) | 308 (2.2) |
|  | Trauma | 1.0 | 731 (5.9) | 817 (5.9) |
|  | Dizziness | 0.9 | 32 (0.3) | 53 (0.4) |
|  | Sinusitis | 0.9 | 867 (7.1) | 960 (6.9) |
|  | Other | 0.9 | 866 (7.0) | 939 (6.7) |
|  | Computed tomography | 20 | 603 (7.0) | 939 (6.7) |
| Neck (cervical spine) | X-ray | 0.1 | 1,508 (12.3) | 1,966 (14.1) |
|  | Computed tomography | 1 | 55 (0.4) | 62 (0.4) |
| Dental | Full mouth | 0.01 | 2,133 (17.4) | 2,622 (18.8) |
|  | Other | 0.002 | 4,730 (38.5) | 4,930 (35.3) |
| Angiography^2^ |  | 5 | 62 (0.5) | 82 (0.6) |
| Sialography |  | 2 | 25 (0.2) | 28 (0.2) |
| Myelogram |  | 0.5 | - | 2 (0.0) |
| Isotope scan | Head | 1 | 2 (0.0) | 2 (0.0) |
|  | Heart | 3 | 11 (0.1) | 9 (0.1) |
|  | Bone | 1 | 12 (0.1) | 10 (0.1) |
|  | Kidney | 1 | - | - |
|  | Thyroid | 1 | 458 (3.7) | 548 (3.9) |
|  | Lung | 0.2 | - | - |

^1^Including skull, facial bones, nasal bones, mandible, temporo-mandibular joint, paranasal sinuses

^2^Including carotid and aortocervical angiography, venography, arteriogram
